# Myricetin: A comprehensive review on its biological potentials

**DOI:** 10.1002/fsn3.2513

**Published:** 2021-08-11

**Authors:** Muhammad Imran, Farhan Saeed, Ghulam Hussain, Ali Imran, Zaffar Mehmood, Tanweer Aslam Gondal, Ahmed El‐Ghorab, Ishtiaque Ahmad, Raffaele Pezzani, Muhammad Umair Arshad, Umar Bacha, Mohammad Ali Shariarti, Abdur Rauf, Naveed Muhammad, Zafar Ali Shah, Gokhan Zengin, Saiful Islam

**Affiliations:** ^1^ Faculty of Allied Health Sciences University Institute of Diet and Nutritional Sciences The University of Lahore Lahore Pakistan; ^2^ Department of Food Science Institute of Home and Food Sciences Government College University Faisalabad Pakistan; ^3^ Neurochemicalbiology and Genetics Laboratory (NGL) Department of Physiology Faculty of Life Sciences Government College University Faisalabad Pakistan; ^4^ School of Life Sciences Forman Christian College (A Chartered University) Lahore Pakistan; ^5^ School of Exercise and Nutrition Faculty of Health Deakin University Burwood Victoria Australia; ^6^ College of Science, Chemistry Department Jouf University Sakaka Saudi Arabia; ^7^ Department of Dairy Technology University of Veterinary and Animal Sciences Lahore Pakistan; ^8^ Endocrinology Unit Department of Medicine (DIMED) University of Padova Padova Italy; ^9^ AIROB Associazione Italiana per la Ricerca Oncologica di Base Padova Italy; ^10^ School of Health Sciences (SHS) University of Management and Technology Johar Town, Lahore Pakistan; ^11^ Department of Technology of Food Productions K.G. Razumovsky Moscow State University of Technologies and Management (the First Cossack University) Moscow Russian Federation; ^12^ Department of Chemistry University of Swabi Swabi Khyber Pakhtunkhwa (KP) Pakistan; ^13^ Department of Pharmacy Abdul Wali Khan University Mardan Pakistan; ^14^ Department of Biology Science Faculty Selcuk University Konya Turkey; ^15^ Institute of Nutrition and Food Science University of Dhaka Dhaka Bangladesh

**Keywords:** anticancer, antidiabetic, *Myricaceae*, Myricetin, phytochemical

## Abstract

Myricetin is a critical nutritive component of diet providing immunological protection and beneficial for maintaining good health. It is found in fruits, vegetables, tea, and wine. The families *Myricaceae*, *Polygonaceae*, *Primulaceae*, *Pinaceae,* and *Anacardiaceae* are the richest sources of myricetin. Different researchers explored the therapeutic potential of this valuable constituent such as anticancer, antidiabetic, antiobesity, cardiovascular protection, osteoporosis protection, anti‐inflammatory, and hepatoprotective. In addition to these, the compound has been tested for cancer and diabetic mellitus during clinical trials. Health benefits of myricetin are related to its impact on different cell processes, such as apoptosis, glycolysis, cell cycle, energy balance, lipid level, serum protein concentrations, and osteoclastogenesis. This review explored the potential health benefits of myricetin with a specific emphasis on its mechanism of action, considering the most updated and novel findings in the field.

## Graphical abstract

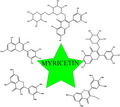

## 1 INTRODUCTION

Natural products might be effective, new, and safe therapeutic agents if properly tested. In the current era, ample of drug molecules have their roots in natural products. The exploration of natural products against various preclinical cell or animal models can afford the emergence of novel drug candidates. Myricetin is found in many plant families including *Myricaceae, Polygonaceae, Primulaceae, Pinaceae,* and *Anacardiaceae* (Abd El‐kader et al., 2013; Borck et al., 2018). It is predominantly present in fruits, vegetables, berries, teas, and wine. Myricetin has been found as free molecule or glycosidically bound such as myricetin‐3‐*O*‐β‐d‐galactopyranoside, myricetin‐3‐*O*‐(4”‐acetyl)‐α‐l‐arabinopyranoside, myricetin‐3‐*O*‐(3”‐acetyl)‐α‐L‐arabinopyranoside, myricetin‐3‐*O*‐α‐l‐rhamnopyranoside, myricetin‐3‐*O*‐β‐d xylopyranoside, myricetin‐3‐*O*‐(6”‐galloyl)‐β‐d‐galactopyranoside, myricetin 3‐*O*‐α‐l‐arabinofuranoside, myricetin‐3‐*O*‐(2”‐*O*‐galloyl)‐α‐l‐rhamnoside, myricetin‐3‐*O*‐(3”‐*O*‐galloyl)‐α‐l‐rhamnoside, and myricetin‐3‐*O*‐α‐l‐rhamnoside (Cao et al., 2018). Myricetin is less hydrophilic (16.6 µg/ml) but has good solubility in basic aqueous and in some organic media such as tetrahydrofuran, dimethylacetamide, acetone, and acetone dimethylformamide (Chang et al., 2012). Moreover, study on the degradation of this compound showed that it is quite stable even at pH 2, but it can differ with temperature. In the late eighteenth century, this compound was isolated from the bark of the *Myrica nagi* Thunb. A light yellow‐colored crystals namely Myricaceae were harvested in India in the past (Perki, 1911). At first, the basic reason behind isolation was its coloring properties. Previously, Perkin (1902) carried out structural and physical properties elucidation and observed it contain various analogs such as ethyl, methyl, bromo, and potassium with melting point of 357°C. Myricetin glycoside (myricetin‐3‐*O*‐rhamnoside) was also found for the first time in this study (Perkin, 1911). Hydrolysis of myricetin resulted in phloroglucinol and gallic acid production, this also resulted to confirm its chemical structure. The structure of the myricetin (**1**) is related to many other phenolic compounds such as quercetin (**3**), morin (**4**), kaempferol (**5**), and fisetin (**6**). Because of its structural similarity, myricetin is also named as hydroxyquercetin (**3**). Literature provides strong evidence about the nutraceuticals and antioxidant properties of myricetin (Lin & Huang, 2012). It also showed many pharmacological activities such as hepatoprotective, antitumour, anti‐inflammatory, analgesic, and antidiabetic. The significant antioxidant activity of myricetin is attributed to the presence of three hydroxyl groups on ring B as compared to other flavonoids. The mineral chelation is attributed to the double‐bonded oxygen group along with the two hydroxyl groups as shown in in vitro studies. Prooxidative effect of the myricetin is due to the catechol groups in its structure which forms semi‐quinone radicals. This radical is oxidized when both 4‐hydroxyl on the B and 4‐hydroxyl group on the C ring form a quinine. There are many health benefits attributed to the myricetin such as inhibited hyperglycemia, decreased hepatic triglyceride, reduced oxidative stress and cholesterol contents, and protected liver injury (Chang et al., 2012; Choi et al., 2014; Guo et al., 2015; Semwal et al., 2016).

**FIGURE 1:**
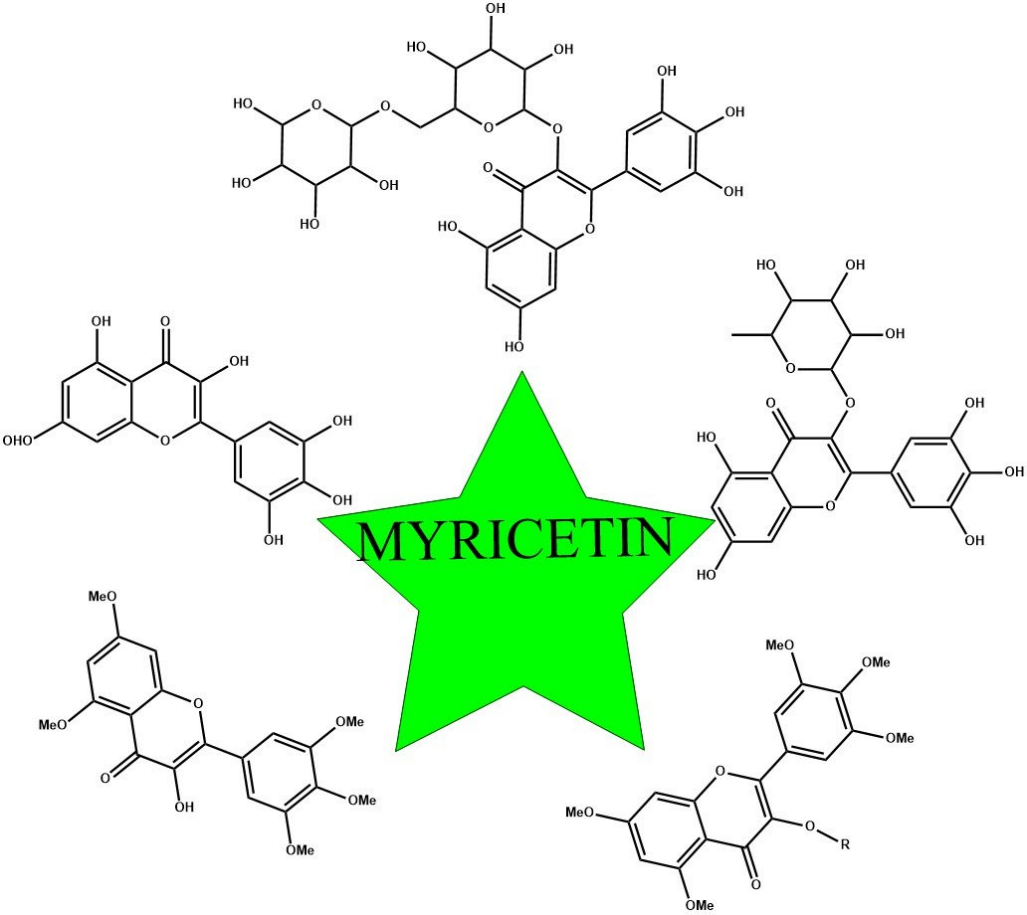
Scheme of myricetin and its derivatives

A study conducted by Dang explored the bioavailability and pharmacokinetic properties of myricetin using ultraperformance liquid chromatography–tandem mass spectrometry method. Myricetin was administrated orally and intravenous to the rats at dose‐dependent manner, later on their bioavailability in blood was also determined (Dang et al., 2014). The results showed that the bioavailability of the myricetin by oral route was less due to the scarce absorption (9.62 and 9.74% at oral doses of 50 and 100 mg/kg, respectively). Maximum concentrations (C max) and area under the cure (AUC) of myricetin increased after oral administration which was proportional to the dose which shows that the myricetin is absorbed by the passive diffusion in vivo. Longer time of achieving maximum concentration (*T*_max_) (6.4 hr) indicated its low aqueous solubility. Pharmacokinetic properties and bioavailability of co‐administered drugs along with myricetin have been studied. The inhibition of the cytochrome P450s (drug metabolizing cytochrome) and P glycoproteins (drug efflux pumps) is a recognized mechanism of myricetin (Li et al., 2011). However, absorption of the myricetin was increased by 41.8%–74.4% and 48.4%–81.7%, respectively, by the combination of myricetin with tamoxifen as compared to the control group in which only tamoxifen was used. Co‐administration of myricetin with losartan was also found to induce metabolism (Choi et al., 2010) (Figure 1).

## 2 HEALTH PROPERTIES

Myricetin possesses interesting pharmacological potentials such as anticancer, antidiabetic, and anti‐inflammatory activities. This paragraph summarizes the existing literature on myricetin offering a comprehensive review on health effects of such molecule. Table 1 reports the fundamental works and the potential mechanisms of action of myricetin (Table 1).

**TABLE 1:** Concentration of myricetin in different food sources (USDA, 2020)

Sources	Amount (mg/100 g)
Blackberry	700
Rutabagas	2,100
Garlic	1,600
Cranberry	6,600
Dock	5,700
Blueberry	1,300
Black berry	700
Peppers, hot chili, green	1,200
Broadbeans, immature seeds	2,600

### 2.1 Anticancer effect

Myricetin has been found a significant inhibitor of migration, invasion, and adhesion and could reduce the matrix metalloproteinase (MMP‐2/9) activities and mRNA levels of ST6 N‐Acetylgalactosaminide Alpha‐2,6‐Sialyltransferase 5 genes (ST6GALNAC5) and MDA‐Mb‐231Br cells in concentration‐dependent manner and in animal model when treated with 50 mg/kg dose (Ci et al., 2018). In recent study by Zhang and colleagues, myricetin induced the suppression of azoxymethane (AOM)/dextran sodium sulfate (DSS) model of (AOM/DSS)‐induced colitis and colorectal tumorigenesis, reduction of size of colorectal polyps, and inflammatory factors IL‐1β, TNF‐α, p‐NF‐κB, IL‐6, NF‐κB, and PCNA (Akhtar et al., 2021; Zhang et al., 2018; Zheng et al., 2017).

Jung et al. (2017) explored that oral administration of myricetin (50 mg/kg/day) in experimental mice for 21 days found increased aerobic capability via enhancing the distance and running time and mitochondrial functions via activating peroxisome proliferator‐activated receptor‐gamma coactivator (PGC‐1α) and SIRT1. The myricetin (25 µM) demonstrated anticancer potential via inducing apoptosis and significantly downregulating the multidrug resistance mutation 1 (MDR‐1) in ovarian cancer cells, A2780 and OVCAR3 (Berköz et al., 2021). A dose‐dependent significant antiproliferative effect has been reported in human papillary thyroid cancer cells (SNU‐790) exhibiting cytotoxicity, inducing DNA condensation, upregulating the Bax: Bcl‐2 ratio, activating caspase cascades, changing the mitochondrial membrane potential, and tempting the release of apoptosis‐inducing factors (Ha et al., 2017). Similarly, myricetin also had a role in lowering the proliferation rate and apoptotic cell death, in modulating cell cycle, invasion and pro‐angiogenic properties via mitogen‐activated protein kinase (MAPK) and PI3K/AKT signaling pathways. In addition, myricetin decreased free radicals, peroxidation of lipids, prevented from depletion of glutathione, and loss of membrane potentials in mitochondria in choriocarcinoma cell models (JEG‐3 and JAR). Moreover, myricetin augmented the cytosolic Ca^2+^ release from the endoplasmic reticulum (ER) linked with ER stress modulation (Yang et al., 2017). Human anaplastic thyroid cancer cell (SNU‐80 HATC) proliferation has also been significantly reduced by the myricetin up to approximately 70%. A concentration‐dependent cell death was observed sub‐G_1_. The myricetin (100 μM) increased in the ratio of the Bax:Bcl‐2 and caspase cascades (Jiang et al., 2019). It was also found that myricetin activated apoptosis through apoptosis‐inducing factors found in the cytosol and mitochondria and by disturbing the membrane potential (Jo et al., 2017). Earlier study reported by Jose et al. found that different doses of myricetin (25 and 100 mg/kg) possessed strong anticancer role against different human lung adenocarcinoma cell line (A549) and human erythroleukemic cell line (K562) (Jose et al., 2016). Oral administration of myricetin one time a day for the period of 84 days in adenomatous polyposis coli multiple intestinal neoplasia (APCMin/+) mice showed a reduction in degree of dysplasia and number of dysplastic cells in each polyp, induction of apoptosis and inhibition of proliferation of cancer cells, modulation of Wnt/β‐catenin and GSK‐3β pathways, reduction of IL‐6 and PGE2 (pro‐inflammatory cytokines) in blood, and downregulation of the p38 MAPK/Akt/mTOR pathways (Li et al., 2016). Anti‐leukemia activity of myricetin was reported by interfering with biosynthetic pathway of purine nucleotides by inhibiting inosine 5'‐monophosphate dehydrogenase (hIMPDH) 1/2 catalytic activity (Mondal et al., 2016). Researchers determined the anticancer role of myricetin against human MCF‐7 cells by applying different doses at (0–80 µM) for 12, 24 and 48 hr (Pujari & Mishra, 2021). Multiple cell pathways were involved with consequent cell viability reduction, apoptosis induction, suppression of protein expression of p21‐activated kinase 1 and phosphorylated extracellular MAPK and activation of Bax protein expression, GSK3β, promotion of caspase‐3 activity, and suppression of β‐catenin and cyclin D1, respectively (Jiao & Zhang, 2016). In another study, myricetin with methyl eugenol (MEG) and cisplatin (CP) in human cervical cancer cells have been found to hinder the growth of cancer cells by inducing apoptosis, dramatically as compared to single drug treatment. The combined treatment showed higher mitochondrial membrane potential loss (ΛΨm) and caspase‐3 activity as compared to single drug treatment (Yi et al., 2015). Myricetin (60 μM) has been tested in human glioma U251 cells, inducing apoptosis, enhancing expressions of Bax and Bad levels, lowering levels of Bcl‐xl and Bcl‐2, and inducing cell cycle arrest in G2/M phase. All of these mechanisms were time‐ and dose‐dependent (Fu et al., 2019). Wang and their colleagues investigated myricetin along with chitosan‐functionalized pluronic P123/F68 micelles. They showed alteration in levels of apoptotic proteins, such as bad, Bcl‐2, and bax, in mice (Rajendran et al., 2021). In another study, myricetin exhibited antiproliferative activity in human cancer cells. In this study, their anti‐angiogenic effects were investigated with in vitro (HUVEC) and in vivo (CAM) models, which showed myricetin inhibited angiogenesis induced by OVCAR‐3 cells. This anticancer effect was attributed to decline secretion of the key angiogenesis mediator vascular endothelial growth factor (VEGF) and decreased levels of p‐Akt, p‐70S6K and hypoxia‐inducible factor‐1α (HIF‐1α) proteins in A2780/CP70 and OVCAR‐3 cells. Transient transfection experiments showed that myricetin inhibited secretion of VEGF by the Akt/p70S6K/ HIF‐1α pathway. Moreover, a novel pathway, p21/HIF‐1α/VEGF, was found to be involved in the inhibitory effect of myricetin on angiogenesis in OVCAR‐3 cells. It also suppressed cell viability, induced nuclear chromatin fragmentation and condensation, upregulated active caspase‐3 protein levels, ER stress related proteins, protein‐78 and C/EBP homologous protein which are glucose‐regulated in a dose‐dependent manner in ovarian cancer cells (SKOV3). Further, apoptosis in SKOV3 cells was also due to myricetin‐induced DNA double‐strand breaks and endoplasmic reticulum stress (Xu et al., 2016) (Tables 2 and 3).

**TABLE 2:** Preclinical diabetic studies of myricetin

Phytochemical	References	Model	Effect
Myricetin	Kandasamy and Ashokkumar (2013)	In vivo	Stimulated lipogenesis in rat adipocytes and increased stimulatory effect of insulin (EC50 = 65 μM)
Myricetin	Kang et al. (2015)	In vivo	Suppressed the α‐glucosidase (IC50 = 414 μM) activity
Myricetin	Pandey et al. (2009)	In vivo	Exhibited antidiabetic activity against t‐BHP‐induced oxidative stress
Myricetin	Kandasamy and Ashokkumar (2013)	In vivo	Increased the enzymatic and nonenzymatic antioxidant defense system in liver and kidney of streptozotocin‐cadmium‐induced diabetic model
Myricetin	Ozcan et al. (2012)	In vivo	Improved and re‐established renal functions and activities of the glutathione peroxidase and xanthine oxidase enzymes in diabetic rat model
Myricetin	Tadera et al. (2006)	In vivo	Suppressed the intestinal α‐glucosidase (29%) and porcine α‐amylase (64%) with IC50 vale of 0.38 mM
Myricetin	Ong and Khoo (2000)	In vivo	Lowered glycemia up to 50% after 2 days of treatment at 3 mg/12 hr
Myricetin	Kandasamy Ashokkumar (2012)	In vivo	Anti‐hyperglycemic and renoprotective effects at 1.0 mg/kg

**TABLE 3:** Health effects of myricetin

Biological effects	Mechanisms of action	References
Anticancer	Inhibited the migration, invasion, and adhesion stages Lowered the MMP−2/9 activities and mRNA levels of ST6GALNAC5	Ci et al. (2018)
	Suppressed the mTOR activation	Cao et al. (2018)
	Suppressed AOM/DSS‐induced colitis and colorectal tumorigenesis Lowered the size of colorectal polyps, inflammatory factors IL−1β, TNF‐α, p‐NF‐κB, IL−6 Decreased the NF‐κB, PCNA, cyclooxygenase−2 (COX−2), and Cyclin D1 expressions	Zhang et al. (2018)
	Enhanced mitochondrial functions via activating PGC−1α and SIRT1	Jung et al.( 2017)
	Induced apoptosis, and significantly downregulated the MDR−1	Zheng et al. (2017)
	Induced DNA condensation, and upregulated the stimulation of Bax:Bcl−2 and caspase cascades the expression ratio	Ha et al. (2017)
	Caused the cell cycle, and attenuated the invasive and pro‐angiogenic properties Lowered peroxidation of lipids Prevented from depletion of glutathione, and loss of membrane potentials Augmented the cytosolic Ca^2+^ release from the endoplasmic reticulum linked with ER stress modulation	Yang et al. (2017)
	Enhanced the ratio of the Bax:Bcl−2 and caspase cascades Ruptured the mitochondrial membrane potential	Jo et al. (2017)
Antidiabetic effects	Significantly enhanced the Nrf2/HO−1 pathway Increased the antioxidant enzymes such as GPx and SOD activities Lowered the MDA production and inhibited the I*κ*B*α*/NF‐*κ*B pathway Lowered the cytokines including TNF‐*α*, IL−6 and IL−1*β* Suppressed the TGF*β*/Smad3 pathway as also noted that regulation of I*κ*B*α*/NF*κ*B by myricetin was independent by suppressing Nrf2 in NRCM for its effect on Nrf2	Liao et al. (2017)
	Enhanced the insulin sensitivity via lowering glucose level	Li et al. (2017)
	Suppressed the α‐amylase and α‐glucosidase activities	Meng et al. (2016)
	Prevented from momentous increment in urea, plasma glucose, uric acid, urinary albumin, blood urea nitrogen, glucose−6‐phosphatase, glycosylated hemoglobin, glycogen phosphorylase, hexokinase, glycogen synthase, and glycogen with insulin signaling molecule expression and fructose−1,6‐bisphosphatase Normalized the insulin signaling molecule expression like PKB (protein kinase B), IRS−1 (insulin receptor−1), IRS−2 (insulin receptor−2), GLUT−2 (glucose transporter−2) and GLUT−4 (glucose transporter−4)	Kandasamy and Ashokkumar (2014b)
Antiobesity effects	Improve hepatic steatosis and systemic insulin resistance along with body weight reduction Modulated thermogenic regulation proteins Enhanced the thermogenic protein expression and beige formation	(Hu et al., 2018).
	Lowered levels of protein expression phosphorylated AKT serine/threonine kinase 1 (Akt)	Yao et al. (2018)
	Up regulated the β‐endorphin and adropin levels	Chao et al. (2017)
Cardiovascular effects	Reduced inflammatory cytokines and inhibited cellular apoptosis Degraded IκBα and nuclear translocation of p65 Prevented from the iNOS overexpression and oxidoreductase activity	Zhang et al. (2018)
	In earlier study, myricetin also has been known to suppress the activator of transcription 1 (STAT1) activation and signal transducer	Scarabelli et al. (2009)
	Normalized the nitric oxide, endothelin nitric oxide synthase, serum high‐density lipoprotein cholesterol, and prostaglandin I2 levels	(Guo et al., 2015).
	Lowered the lactate dehydrogenase and creatine kinase levels Reduced the infarct size and cardiomyocyte apoptosis levels Reduced levels of MDA and increased levels of GSH/GSSG ratio Downregulated the p38, cytochrome P450, and cyclooxygenase−2 Upregulated fatty acid synthase and 6‐phosphogluconate dehydrogenase	Qiu et al. (2017)
Anti‐inflammatory effects	Decreased the keratinocyte death, and COX2, IκB/NFκB expressions	Xie and Zheng (2017)
	Suppressed the NF‐κB, IL−6–12, NO, iNOS, TNF‐α (inflammatory mediators), binding activity NF‐κB DNA Altered the NF‐κB, IκBα, phosphorylation of STAT1 and production	Cho et al. (2016); Latief et al. (2015)
	Suppressed the pro‐inflammatory mediators, that is, TNF‐α‐stimulated creation, Akt, mTOR and NF‐κB	Lee and Lee (2016)
Hepatoprotective effects	Lowered the AST and ALT levels Regulated by the myricetin such as caspase−3/9 and P53 protein, mitogen‐activated protein kinase, nuclear factor‐kappa B (NF‐κB) activation inhibition of toll‐like receptor 4 (TLR4), heme oxygenase−1 (HO−1), increase and enhanced expression of Nrf2 (nuclear factor‐erythroid 2‐related factor 2) Lowered AMPK/ACC signaling and activated Keap1‐Nrf2/HO−1 Enhanced the HO−1 and Nrf2 protein expressions	Lv et al. (2020)
	Hampered phosphorylation of Smad2, type I deposition by suppression of α‐smooth muscle actin and collagen	Geng et al. (2017)
	Lowered miR−146b expression to elevate TRb levels	Xia et al. (2019)
Osteoporosis prevention	Increased body weight gain, upregulated osteocalcin Improved alkaline phosphate activity, and inhibited reduction Lowered tartrate‐resistant acid phosphatase and C‐terminal telopeptide of type I collagen levels Enhanced the osteopontin (OPN) levels, collagen type I alpha 1, COL1A1, ALP, Runx2, BMP2 and OCN	Fan et al. (2018)
	Prevented from bone resorption Increased alveolar rest height	Huang et al. (2016)

Myricetin weakened the cancer cells neoplastic transformation by interacting with oncoproteins such as protein kinase B (PKB) (Akt), Fyn, Janus kinase‐signals and activation of JAK1‐STAT3 MEK1. Additionally, myricetin also has antimitotic effects which is attributed to the control of the overexpression of kinase 1 (CDK1) which is cyclin‐dependent and by targeting the mitochondria deaths resulting in the various type of cell death. In a study conducted on two cisplatin‐resistant ovarian cancer cell lines, A2780/CP70 and OVCAR‐3, vascular endothelial growth factor reduced the secretion of important angiogenesis mediator such as hypoxia‐inducible factor‐1α (HIF‐1α), p‐Akt and p‐70S6K protein levels (Huang, Chen, Rojanasakul, et al., 2015; Huang, Chen, Ye, et al., 2015). In another study by Feng and colleagues, cell cycle arrest, induction of apoptosis and reduction of cell proliferation as well as regulation of related proteins GC HGC‐27 and SGC7901 cells were observed after myricetin treatment. The phytochemical also exhibited strong binding affinity for RSK2 that resulted in a prompt expression of the Mad1 (Feng et al., 2015). In hepatocellular carcinoma cell line, myricetin suppressed the p21‐activated kinase 1 (PAK1) in Ras signaling pathway and activated intrinsic caspase‐mediated apoptosis. This resulted in reduced expression of survivin and anti‐apoptotic Bcl‐2, as well as pro‐apoptotic Bax increase. MAPK/ERK and PI3K/AKT signaling pathways were blocked along with downstream Wnt/β‐catenin pathway (Iyer et al., 2015).

### 2.2 Antidiabetic effects

Since it is an antidiabetic agent, myricetin (200 mg/kg/day) was found to be effective in the treatment of mice having dilated cardiomyopathy (DCM)‐associated cardiac injury. In this trial, significant alleviation in apoptosis interstitial fibrosis and cardiac hypertrophy was observed during 6‐month treatment. Also, significant stimulation of Nrf2/HO‐1 pathway was demonstrated. Similarly, GPx and superoxide dismutase (SOD) activities were reversed, and malondialdehyde (MDA) production diminished resulting in increased antioxidative stress capacity. In addition, inflammation process was modulated, given the inhibition of I*κ*B*α*/NF‐*κ*B pathway that resulted in cytokines (TNF‐*α*, IL‐6, and IL‐1*β*) decrement. Consistently, TGF*β*/Smad3 pathway was suppressed in DCM mice treated with myricetin. Bax and caspase‐3 decrements showed the advantageous effects of myricetin treatment on cardiomyocytes. Neonatal rat cardiomyocytes (NRCM) treated with high glucose have also been tested using myricetin with similar results. It was also noted that regulation of I*κ*B*α*/NF*κ*B by myricetin was independent by suppressing Nrf2 in NRCM (Liao et al., 2017). Another work explored the potential effects of myricetin as a natural GPCR (G protein‐coupled receptor) agonist to treat T2DM (type 2 diabetes mellitus). The possible mechanism associated with GPCR agonistic effect of myricetin is the stimulation of secondary messenger (cAMP), which further triggers protein kinase A/C (PKA/PKC) and then transcriptional factors (TF) are activated (TFa) the TFa then internalized to the nucleus and producing special mRNA with special protein coding. Once the mRNA is released to the cytoplasm, initiating the protein synthesis, these special proteins are transplanted act the cell surface in the form of glucose transporters (Glut) which stimulate the glucose uptake. This glucose uptake is responsible for hypoglycemic actions. Glucagon‐like peptide‐1(7–36) amide (GLP‐1) is a secreted peptide that acts as a key determinant of blood glucose homeostasis by virtue of its abilities to slow gastric emptying, to enhance pancreatic insulin secretion, and to suppress pancreatic glucagon secretion. Glucagon‐pike peptide‐1 (GLP‐1) is secreted from enteroendocrine cells (L cells) of the gastrointestinal mucosa in response to a meal, and the blood glucose‐lowering action of GLP‐1 is terminated due to its enzymatic degradation by dipeptidyl‐peptidase‐IV (DPP‐IV). Released GLP‐1 activates enteric and autonomic reflexes while also circulating as an incretin hormone to control endocrine pancreas function (Li et al., 2017). The supplementation of myricetin (770 μg/ml) prevented the postprandial hyperglycemia through inhibiting the α‐amylase and α‐glucosidase activities (Meng et al., 2016).

Cadmium with streptozotocin (STZ) induce diabetic nephrotoxicity in rats which showed enhancement in the concentrations of urinary albumin and indicators of lipid profile like total cholesterol, low density lipoprotein (LDL), triglycerides and decline in the concentration of HDL. Similarly, activities of lecithin cholesterol acyl transferase and lipoprotein lipase were reduced, whereas 3‐hydroxy 3‐methylglutaryl coenzyme A (HmgCoA) reductase activity was enhanced by STZ along with sterol regulatory element‐binding protein (SREBP‐1a, SREBP‐2, and SREBP‐1c), transformation in TGF‐β1 growth factor, and downregulation of PPAR‐α (peroxisome proliferator‐activated receptor). On the other side, experimental subjects treated with 1.0 mg/kg body weight myricetin for the period of 12 weeks reverted these changes. Additionally, glomerulosclerosis and interstitial fibrosis and extracellular mesangial matrix suppression were reported after myricetin treatment (Kandasamy & Ashokkumar, 2014a). A study conducted by Kandasamy and Ashokkumar found that experimental rats were treated with myricetin and prevented from momentous increment in urea, plasma glucose, uric acid, urinary albumin, blood urea nitrogen, glucose‐6‐phosphatase, glycosylated hemoglobin, glycogen phosphorylase, hexokinase, glycogen synthase, and glycogen with insulin signaling molecule expression and fructose‐1,6‐bisphosphatase and a significant decrease of plasma hemoglobin, insulin, glucose‐6‐phosphate dehydrogenase, creatinine. Moreover, myricetin also normalized the insulin signaling molecule expression like PKB (protein kinase B), IRS‐1 (insulin receptor‐1), IRS‐2 (insulin receptor‐2), GLUT‐2, and GLUT‐4 (Kandasamy & Ashokkumar, 2014b). A group of researchers and investigators found that intraperitoneal administration of myricetin (6 mg/day) caused reductions in glomerulosclerosis, urinary volume, BUN, and protein excretion in diabetic male albino Wistar rats tempted by 50 mg/kg injection (intraperitoneal) of streptozotocin. Additionally, restoration of changed renal actions of glutathione peroxidase and xanthine oxidase (Ozcan et al., 2012), whereas Pandey et al. (2009) found that myricetin also lowered the malondialdehyde and protein carbonyl contents in diabetic erythrocytes. Multiple studies involved in protecting role of myricetin in insulin‐resistant rats. In another study, 1mg/kg body wt. of myricetin was injected three times a day for the period on 14 days. The plasma glucose level of the rats starts to normalize which have been previously made high by feeding on high fructose diet along with an increase in plasma β‐endorphin. On the other hand, this treatment also resulted in increase of HOMA‐IR index which can be reduced by the β‐funaltrexamine hydrochloride (β‐FNA) administration. The dose level is adjusted to block MOR (μ‐opioid receptors). Akt and Akt substrate of 160 kDa, insulin receptor substrate‐1, and phosphorylation of the insulin receptor were also found to be affected by the myricetin treatment which ultimately has effect on glucose transporter subtype 4 translocation which were blocked by β‐FNA pretreatment (Tzeng et al., 2011).

**FIGURE 2:**
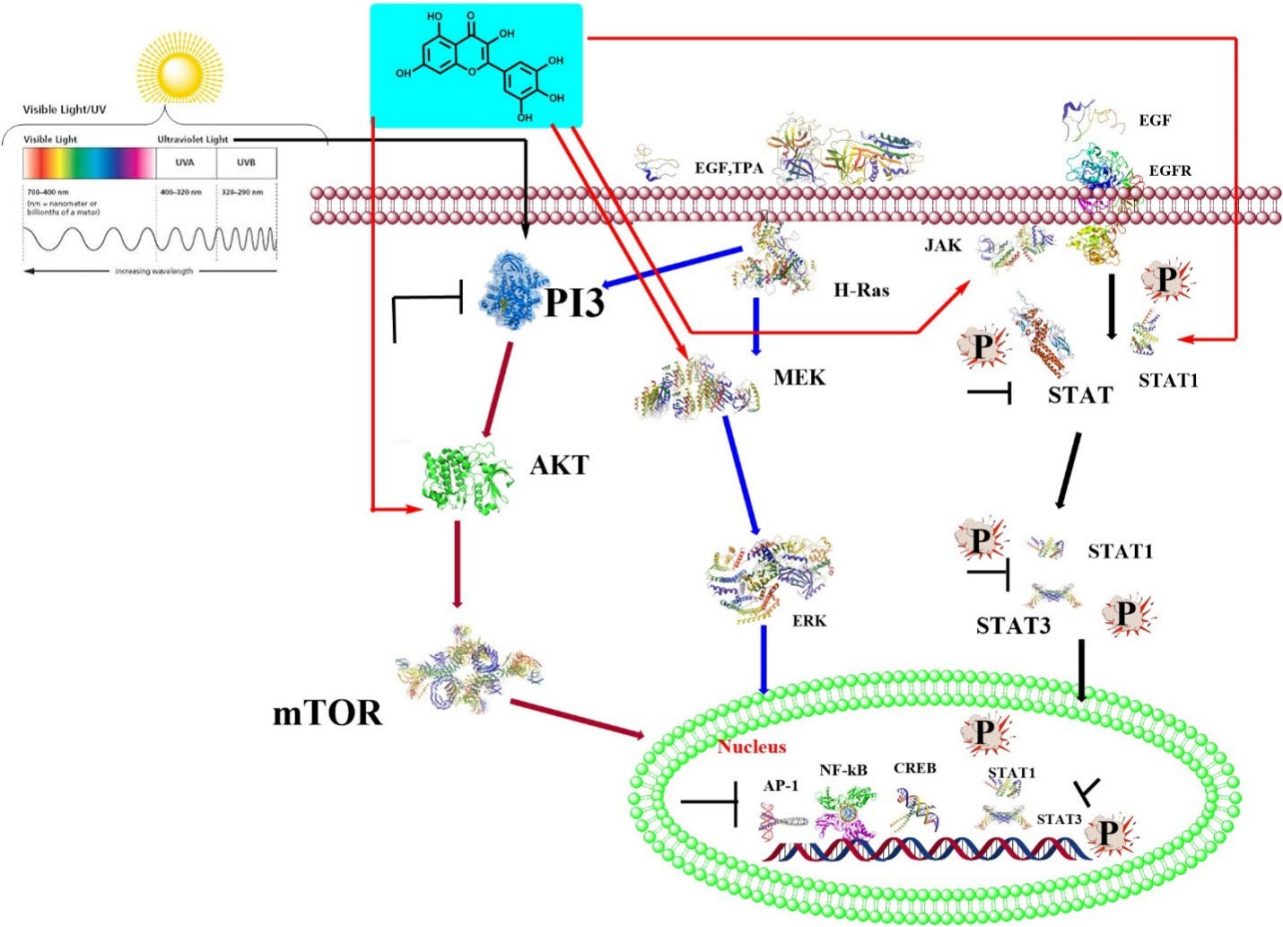
Scheme of myricetin anticancer signaling pathway

The effect of myricetin was evaluated in diabetes mellitus‐associated kidney injuries and dysfunction in an experimental mouse model with diabetes mellitus induced by five consecutive injections of low‐dose streptozotocin (STZ). The data revealed that myricetin (orally twice a day, 100 mg/kg/day, for six moths) inhibited the IκBα/NF‐κB pathway, with this pathway being independent of nuclear factor‐erythroid 2‐related factor (Nrf2) regulation. It was also reported that myricetin activates glucagon‐like peptide‐1 receptor (GLP‐1R) and its long‐term oral administration (200 mg/kg, for 40 days) validates its glucoregulatory effects (Taheri et al., 2020) (Figure 2).

### 2.3 Antiobesity effects

A significant antiobesity effects of myricetin have been reported by different researchers. In a work on mice brown adipose tissues, myricetin supplementation (400 mg/kg) in diet resulted in significant improvement of hepatic steatosis and systemic insulin resistance along with body weight reduction. This reduction in weight resulted ameliorated lipid plasma levels, increased energy expenditures, and decreased adiposity. The main mechanism behind the antiobesity properties of myricetin can be considered the ability to modulate thermogenic regulatory proteins. Indeed, brown adipose tissue (BAT) was activated which in turn activates the mitochondrial biogenesis producing body heat. Other mechanisms include increased thermogenic protein expression and beige formation. It was also found that administration of myricetin at the time of differentiation in C3H10T1/2 (mouse fibroblast) cells resulted in thermogenic gene expression regulated by myricetin. Moreover, adiponectin was increased in these cells (Hu et al., 2018).

In mouse adipocyte 3T3‐L1 cells, myricetin significantly reduced phosphorylated Akt levels (Yao et al., 2018). Reduction in feed efficiency, weight gain, adipocyte size, weight and size of the epididymal and perirenal adipose tissues, level of blood lipids, as well as upregulation of β‐endorphin and adropin levels were reported after myricetin treatment in obese male Sprague‐Dawley rats (Chao et al., 2017). C57BL/6 mice, fed on high‐fat diet were treated with Myricetin, triggering momentous alleviation in steatosis, decrease in thiobarbituric acid reactive substance (TBARS) levels, hepatic lipid accumulation, enhancement in antioxidative enzyme activities.

Moreover, protein expression of NAD(P)H quinone dehydrogenase1 and heme oxygenase‐1, resulted in PPARγ protein expression reduction and increased in nuclear hepatic Nrf2 translocation (Xia et al., 2016). Another study on C57BL/6 mice fed on high‐fat diet found that reduction in body weight, serum glucose, cholesterol, & triglyceride, malondialdehyde and TNF‐α (tumor necrosis factor‐α), enhancement in GPX (glutathione peroxidase) activity, expression of PPARγ (adipogenic transcription factors), downregulation of C/EBPα (CCAAT/enhancer‐binding proteinα), antioxidant capacity and lipogenic transcription factor SREBP‐1c was obtained when animals were treated by myricetin (Su et al., 2016).

Similarly in C57BL/6J male mice, myricetin had the ability of reducing obesity and insulin resistance. High sucrose diet was used to induce obesity in mice followed by the administration of the 0.12% myricetin. The compound significantly affected all the markers of obesity such as weight gain, epididymal white adipose tissue, hypercholesterolemia, hypertriglyceridemia. HOMA‐IR values along with insulin level and serum glucose decreased significantly during the trial. Also, leptin, IL‐6, and TNF‐α levels were also reduced in the serum (Choi et al., 2014).

In another work, myricetin downregulated mRNA and adipogenic transcription factors and suppressed the differentiation of 3T3‐L1 mouse preadipocytes, along with decrease level of sterol regulatory element‐binding protein 1‐c in mRNA and lipoprotein lipase and glucose transporter‐4. Furthermore, enhancement of Jun N‐terminal kinase and phosphorylation level of Erk and p38 were observed during lipolysis, together with a downregulation (in a dose‐dependent manner) of perilipin A mRNA level (Wang et al., 2015).

A study was conducted to explore the hepatoprotective potential of myricetin against HFD (high‐fat diet)‐induced hepatic steatosis C57BL/6 mice, and then, the rats were treated with 0.12% w/w myricetin in the diet. Myricetin significantly reduced HFD‐induced steatosis, TBARS levels, and lipid accumulation along with showed enhancement in SOD, glutathione peroxidase (GPx) and catalase (CAT) antioxidant enzyme activities in the liver. Myricetin is involved in the liver nucleus Nrf2, NAD(P)H quinone dehydrogenase 1 (NQO1) and heme oxygenase‐1 (HO‐1) stimulate increased production of more protein, PPARγ induces a decrease in protein expression, and genes are involved in the PPAR signaling pathway and peroxidation. Proteins in the bioenzyme pathway show standardized expression (Choi et al., 2014).

Myricetin inhibited YAP (YES‐associated protein) expression by stimulating kinase activation of LATS1/2. Knockdown expression of LATS1/2 by shRNA attenuated myricetin‐induced phosphorylation and degradation of YAP. Furthermore, myricetin sensitized (hepatocellular carcinoma) HCC cells to cisplatin treatment through inhibiting YAP and its target genes, both in vitro and in vivo. The identification of the LATS1/2‐YAP pathway as a target of myricetin may help with the design of novel strategies for human HCC prevention and therapy (Lv et al., 2020).

Myricetin effectively protected from LPS/D‐GalN‐induced fulminant hepatitis by lowering the mortality of mice, decreasing ALT and AST levels and alleviating histopathological changes, oxidative stress, inflammation and hepatic apoptosis. In vitro study suggested that myricetin remarkably attenuated H2O2‐triggered hepatotoxicity and ROS generation and activated Keap1‐Nrf2/HO‐1 and AMPK/ACC signaling pathway (Li et al., 2019).

### 2.4 Cardiovascular effects

Oxidative stress and inflammation are usually linked with the signs of complication associated with myocardial dysfunction induced by sepsis. Inflammation induced by 10 mg/kg lipopolysaccharide (LPS) in mice has been treated by 100 mg/kg myricetin, showing reduction in inflammatory cytokines in cardiac tissues as well as in serum. Myricetin inhibited cellular apoptosis, degradation of IκBα and nuclear translocation of p65, with prevention of iNOS overexpression and oxidoreductase activity. Peritoneal macrophages stirred by LPS in vitro can produce inflammatory cytokines other than myricetin treatment (Zhang et al., 2018). In ischemia/reperfusion (I/R) treated rats, myricetin (5 μM) treatment resulted in increase in the left ventricular pressure, improved coronary flow and maximum up/down pressure development in the left ventricular, decrease lactate dehydrogenase and creatine kinase levels in coronary flow. Moreover, infarct size and cardiomyocyte apoptosis levels were also reduced by myricetin, with reduced MDA amount. Increased levels of SOD and GSH/GSSG ratio showed that myricetin has antioxidant properties. Myricetin activity on signaling pathways seems to be related to the downregulation of p38, cytochrome P450 and cyclooxygenase‐2 as well as the upregulation of fatty acid synthase and 6‐phosphogluconate dehydrogenase (Qiu et al., 2017). In previous study, myricetin suppressed the activator of transcription 1 (STAT1) activation and signal transducer (Scarabelli et al., 2009). Induction of choline to mice significantly enhanced the concentration of total triglyceride, low‐density lipoprotein cholesterol, total serum cholesterol, thromboxane A2, endothelin 1, alanine, as well as TXA2 levels, aspartate aminotransferase activities and aminotransferase. Choline also lowered NO, eNOS, serum HDL cholesterol and prostaglandin I2 levels whereas administration of myricetin (400 and 800 mg per kg bw) to experimental mice normalized these changes (Guo et al., 2015). Subcutaneously isoproterenol given two time a day at a dose of 85 mg/kg along remarkably enhanced the concentration of cardiac marker enzymes, that is, lactate dehydrogenase, creatine kinase and aspartate aminotransferase in serum, and lowered the levels of catalase and dismutase: when myricetin was given at a dose ranging from 100 to 300 mg/kg p.o., it reverted these changes in animals (Tiwari et al., 2009). Chen and collaborators determined that myricetin provided protection against LPS‐induced cardiomyocyte injury and suppressed NF‐κB/P65 signaling pathway (IL‐6, TNF‐α, IL‐1β markedly reduced). Moreover, elevated levels of glutathione peroxidase and SOD were observed as a result of decrease in the concentration of ROS. The anti‐inflammatory and antioxidant effects of myricetin are significant but the effect on lipopolysaccharide (LPS)‐induced cardiac injury remains to be tested more. This study aimed to explore whether myricetin was efficient to alleviate SIMD in mice and neonatal rat cardiomyocytes injury. Mice administrated with myricetin (100 mg/kg, po, bid) or vehicle groups were challenged with LPS (10 mg/kg, ip), and cardiac functions examined by echocardiography after 12 hr LPS exposure. LPS markedly impaired mouse cardiac functions, which were significantly attenuated by myricetin administration. Myricetin significantly reduced the production of inflammatory cytokines both in serum and cardiac tissue. Myricetin could inhibit the nuclear translocation of p65, degradation of IκBα, and cellular apoptosis in vivo and in vitro. Myricetin also prevented overexpression of iNOS and reduction of oxidoreductase (SOD and GPx) activity. Besides, Myricetin treatment could attenuate production of inflammatory cytokines of peritoneal macrophages stimulated with LPS in vitro. Thus, we concluded that myricetin could attenuate the LPS‐induced cardiac inflammation injury in vivo and in vitro. Myricetin may be a potential therapy or adjuvant therapy for Sepsis induced myocardial dysfunction (Chen & Fan, 2018).

### 2.5 Anti‐inflammatory effects

The COX‐I is distributed throughout the body tissues as compared to COX‐II, moreover the COX‐II is over expressed at the site of inflammation as compared to COX‐I. The suppression or blockage of COX‐I/COX‐II can suggest analgesic, antipyretic and anti‐inflammatory effects. The suppression of COX‐II produced by myricetin could indicate its anti‐inflammatory potential. Studies on myricetin administration on human keratinocytes showed that TNF‐α stimulated creation in inflammatory mediators. However, these mediators are still not well studied. There are different pathways such as Akt/mTOR and NF‐κB involved in gene transcription in response to the inflammatory stress. These pathways are now well explored in human keratinocytes (HaCaT) cells (Lee & Lee, 2016). It is revealed from the studies that TNF‐α‐stimulated inflammatory mediator making is reduced by deactivation of the several other metabolic pathways including NF‐κB, mTOR and Akt. Production of the reactive oxygen is actually inhibited by the induction of the myricetin. Moreover, myricetin also proved to change skin inflammatory disease by suppressing the pro‐inflammatory mediators. Another study showed that myricetin could inhibit NF‐κB, IL‐6, IL‐12, NO, iNOS, TNF‐α (inflammatory mediators), could modulate the binding activity of NF‐κB to DNA and degradation of the p65 NF‐κB subunit, phosphorylation of STAT1 in LPS‐ and IFN‐β‐stimulated RAW264.7 macrophages. Similarly, Nrf2/HO‐1 system resulted modified by the use of myricetin (Cho et al., 2016; Latief et al., 2015).

A study on the iNOS in C57B16/J knockout male Swiss mice (iNOS(−/−)) treated by myricetin resulted in significantly lowered production of NO in the paws of tested animals. Also, paw edema induced by the carrageenan was reduced as compared to the iNOS(−/−) mice. In addition, myricetin was shown to possess antinociceptive effect and this ability was not reversed by the pre‐administration of naloxone. The antinociceptive effect is related to the antagonist effect on COX, but when naloxone inhibited the antinociceptive effect, means that the myricetin might be an agonist for opioids receptors like GPCR. The agonistic effect of myricetin on opioids receptors leading to the decrease in the concentration of cAMP, which is responsible for the inhibition of PKA caused closing of the calcium channel resulting lower intracelluler calcium concentration. This decline of intracellular calcium hindering the release of neurotransmitters especially substance P, which are responsible for central analgesic effect. It was concluded that the anti‐inflammatory and antinociceptive effects of myricetin could be imputable to the inhibition of iNOS (de Oliveira Azevedo et al., 2015).

The main reason behind progressive loss of β‐cell is the cell death induced by the Cytokine. Series of events are triggered by the IFN‐γ (interferon γ), IL‐1β (interleukin) and TNF‐α (tumor necrosis factor α) which ultimately lead to β‐cells death. Use of the myricetin as anticytoprotective and inflammatory mediator has increased in the recent past. A study conducted using insulin‐secreting RIN‐m5f β cells demonstrated the protective effect of myricetin at 10 μM and 20 μM, that minimized cell apoptosis due to the inflammatory effects induced by TNF‐α and increased cell viability for 3 days. Moreover, myricetin especially at 20 μM affected NFκB factor (by increasing the total and p65 subunit levels), mitochondrial release of cytochrome c increase, NO accumulation, production of ROS (Ding et al., 2012).

Human gingival fibroblasts (HGF) are characterized by the reduced expression of enzymes activity such as MMP‐2, MMP‐8 and MMP‐1. Formation of the NF‐κB ligand (RANKL)‐stimulated TRAP(+) multinucleated cells activator could be inhibited by myricetin. In the RAW264.7 cells, myricetin blocked ERK and cSrc signaling, RANKL‐stimulated activation of p‐38 and degradation of I(k)B. Similarly, NFATc1 transcription factors induced RANKL induction was blocked by myricetin. In RAW264.7 cells myricetin reduced the expression of genes associated with the mRNA osteoclast‐associated genes including TRAP, cathepsin K and cFOS, together with inhibition of LPS‐induced IL‐1β and TNF‐α (Ko, 2012).

Mouse bone marrow (LPS‐stimulated)‐derived dendritic cells (DCs) treated with 10 µg/ml myricetin resulted in a significant reduction in TNF‐α secretion, IL‐12 and IL‐6. Histocompatibility CD40, CD86 and class II was also blocked by the flavonoid as well as migratory and endocytic capacity of DC cells. Moreover, T‐cell proliferation by LPS‐stimulated DC‐elicited allogene was abolished by myricetin (Fu et al., 2019; Hagenacker, Hillebrand, Büsselberg, et al., 2010; Hagenacker, Hillebrand, Wissmann, et al., 2010). Different works on IL‐1β‐stimulated SW982 synovial cells proved anti‐inflammatory role of myricetin along with the reduction in production of MMP‐1 and IL‐6, weakening the phosphorylation of p38 MAPK and Jun NH2‐terminal kinase (Lee & Lee, 2016).

### 2.6 Hepatoprotective effects

Hepatoprotective effect of myricetin is well documented by different researchers all over the world. D‐GalN (D‐galactosamine) and LPS‐induced fulminant hepatitis: myricetin as proved by the lower mortality rate in animals could reduce such inflammation process. Other markers such as AST and ALT levels were also decreased along with reduced oxidative stress, hepatic apoptosis, histopathological changes and inflammation. In addition, several metabolic key factors were mediated and regulated by myricetin, including caspase‐3/9 and P53, MAPK, NF‐κB, toll‐like receptor 4 (TLR4), HO‐1, Nrf2, acetyl‐CoA carboxylase (ACC) and AMP‐activated protein kinase (AMPK). Most importantly in hepatotoxicity induced by H_2_O_2,_ AMPK/ACC signaling and activated Keap1‐Nrf2/HO‐1 were reduced by myricetin administration. On the other hand, HO‐1 and Nrf2 protein expression was enhanced and reversed by AMPK and Nrf2‐null inhibitor (Lv et al., 2020). In a recent study Xia et al. found that myricetin (100 mg/kg oral administration for 16 weeks) regulated the lipid metabolism and thyroid hormone (TH). The trial was conducted on the C57BL/6J high‐fat diet‐induced hepatic steatosis mice. Other effects included increase in the level of serum thyroid, hepatic type 1 deiodinase (Dio1), energy expenditure, improved hepatic steatosis and inhibition of miR‐146b and miR‐205 upregulation. miR‐205 did not affect mRNA of Dio1 owing to the knockdown or overexpression in primary mouse hepatocytes. Mice treated with miR‐146b mimic‐treated hepatocytes showed decrease in miR‐146b expression to elevate TRb levels by myricetin. TRb siRNA in free fatty acid (FFA) hepatocytes treatment however could abolish the beneficial effect on hepatic thyroid and lipid metabolism (Xia et al., 2019). In another in vitro study, liver fibrosis was induced in the hepatic stellate cell (HSC) line CFSC‐8B by the platelet‐derived growth factor BB (PDGF‐BB) or by TGF‐β1. It was proved that PDGF‐BB or TGF‐β‐induced HSCs activation was abolished by myricetin, that also modulated cell migration, production of extracellular matrix, phosphorylation of TGF‐β1, P38, Smad2, ERK, protein kinase B, Akt in a dose‐dependent manner. Antifibrosis role of myricetin have also been studied on the carbon tetrachloride (CCl_4_) mouse model. The results suggested that the polyphenol could block phosphorylation of Smad2 type I deposition by suppression of α‐smooth muscle actin and collagen, protein kinase activated with mitogen and Akt in CCl_4_ treated mice. The author claimed that myricetin could be potentially used as therapeutic agent against liver fibrosis (Geng et al., 2017).

### 2.7 Osteoporosis prevention

The beneficial effect of myricetin against osteoporosis could be due to its inhibitory effect on osteoclastogenesis and elevation of osteogenic differentiation. In in vivo and in vitro study on glucocorticoid induced osteoporosis (GIOP), the potential effect of myricetin was tested using male Sprague‐Dawley rats. Animals were treated both with at 0.1 mg/kg of dexamethasone and 1 or 2.5 mg/kg myricetin for 35 days. During the trail body weight, bone mineral density, levels of bone turn over and histological changes were investigated. Similarly, dexamethasone (1 μM) and myricetin (20 μM) were used in MC3T3‐E1 cells, evaluating mineralization, differentiation and osteoblast proliferation. In in vivo experiments, the use of myricetin resulted in body weight gain, upregulation of osteocalcin, improve of alkaline phosphate activity, reduction in bone mineral density, bone morphogenetic protein 2 and related TF‐II. Likewise, TRAP (tartrate‐resistant acid phosphatase) activity and CTx (C‐terminal telopeptide of type I collagen) levels were reduced. Moreover, histological changes in the femurs were also ameliorated by the myricetin. Results of the in vitro experiments revealed improved mineralization in DEX‐treated MC3T3‐E1 cells and osteoblast differentiation by myricetin along with increase in osteopontin (OPN) levels, collagen type I alpha 1, COL1A1, ALP, Runx2, BMP2 and OCN (Fan et al., 2018).

Effects of myricetin have been also implicated in osteoclastogenesis protection by affecting the metabolism, cell signaling and cytokines. Ovariectomized (OVX) mouse model was used to investigate the effect on alveolar bone loss by myricetin along with in vitro study on osteoclast formation and bone resorption. C57BL/J6 female mice (*n* = 24) aged eight weeks were randomly divided into four groups. Both low and high dose of the myricetin proved to be helpful for the prevention of bone resorption, increased alveolar crest height and prevention of alveolar bone resorption (Huang et al., 2016).

In human derived cells, treatment with myricetin of bone marrow stem cells (hBMSCs) improved mineralization process, ALP activity and its expression, Runt‐related transcription factor 2 (RUNX2), osteocalcin (OCN) and collagen type I. Moreover lymphoid enhancer factor‐1 (LEF‐1) and T‐cell factor‐1(TCF‐1) of the Wnt/β‐catenin pathway were modulated by the polyphenol, with reduction of β‐catenin (Ying et al., 2014). Inhibition of ERK and c‐Src signaling, inhibited the RANKL‐stimulated degradation of I(k)B in the RAW264.7 cells and inhibition of the RANKL‐stimulated activation of p‐38 cell was supported by myricetin treatment. Moreover, revoking of some transcription factors such as RANKL‐stimulated induction of NFATc1 was modulated by myricetin. Decreased expression of mRNA by myricetin was also noted for osteoclast‐associated genes, TRAP and cathepsin K in the RAW264.7 cells. In the same cell line myricetin inhibited the release of LPS‐induced IL‐1β and TNF‐α (Ko, 2012).

### 2.8 Other properties

Several therapeutic effects can be associated with the use of myricetin. One of them is its beneficial effect against cataract development thank to the strong aldose reductase inhibition, as proved in a study conducted on the galactosemic rats by using 1% dose of the myricetin (Mohan et al., 1988). Hodges and coworkers showed that in an in vivo study a dose of 1 mg/kg, i.v. could the intraocular pressure in normotensive rabbits, suggesting as myricetin was noticeably beneficial for treatment of glaucoma (Hodges et al., 1999). Similarly, in human retinal pigment epithelial cells, myricetin at different levels (10, 20, 50 and 100 µM) reduced cell proliferation and migration, as well as the Vascular endothelial growth factor (VEGF) secretion (Chen et al., 2014). At low concentration, myricetin reduced VEGF expression whereas at high concentration increased VEGF. Similarly, high doses affected cell viability inducing necrosis. Myricetin could also trigger caspase‐3 independent retinal pigment epithelial cell necrosis by the activation of phospholipase A2 and calpain as well as by producing free radicals. Myricetin seems to have a role also in coagulation cascade and platelet aggregation. In rabbit platelets, Zang et al. (2003) found inhibitory effect of myricetin on specific receptors bindings of PAF (platelet activating factor). Myricetin concentration at 1, 2 and 4 nM resulted in 34.8, 85.7 and 119 µM IC50 values for [3H] PAF, respectively. IC50 of 13.1 µM of myricetin was found for the rabbit platelet adhesion. The polyphenol was also effective on neutrophil elastase and thrombin activity, with IC50 values of 7 and 28 µM, respectively (Melzig & Henke, 2005). Different IC50 values such as 17.5–64.1 µM inhibited the agglomeration and release of PAF‐induced serotonin. Contrarily, lower concentration (7.9 µM) possessed no effect on the release of serotonin from platelets (Chen et al., 2001). Another research showed that myricetin could hamper thrombin production which made it very useful in the thrombotic disease treatment (Liu et al., 2010). Increased levels of platelet adenosine 3’,5’‐cyclic monophosphate (cyclic‐AMP) stimulated by prostacyclin was triggered by myricetin. The detailed mechanism behind the anti‐aggregating activity was the modification in the metabolism of platelet cyclic‐AMP followed the phosphodiesterase activity inhibition.

A study conducted on cat blood showed that platelet aggregation was stopped by the intravenous administration of the myricetin at 3.6 µg/kg body weight. Moreover in in vitro experiment 60 nM of myricetin could disaggregate platelet thrombi. Myricetin could bind to platelet membranes and inhibit the formation of prostacyclin synthase reducing radicals of the oxygen, for example, singlet oxygen, hydroxyl radical (¨OH), superoxide anion radical ($O_{2}^{\cdot \cdot }$) and perhydroxyl radical (HO_2_). In addition, PGE2 levels in peritoneal fluid (reduced by myricetin) were decreased, eliciting less platelet aggregation (Tong et al., 2009).

Also, neuroprotective potential of myricetin was explored. A rat model was used to investigate the effect of 0.1–10 mg/kg i.p. myricetin for its remarkable effect on the neuropathic pain. Thermal hyperalgesia and mechanical allodynia were induced by reducing spinal nerve ligation for several hours (Hagenacker, Hillebrand, Büsselberg, et al., 2010; Hagenacker, Hillebrand, Wissmann, et al., 2010). They showed 18%–78% decrease of IK(V) in an in vitro study by using nerve cell at concentration pf 1–75 µM. Reduction in the IK(V) was found to be dependent on p38 modulation. Significant analgesic effect was produced by myricetin as proved by the writhing test induced by acetic acid and time to lick during the late phase of formalin test. A 10–100 mg/kg i.p. dose of myricetin in the mice's hind paws was experimented in bradykinin‐induced nociception assay. The dose of 100 mg/kg provoked a 57% reduction in the cinnamaldehyde‐induced nociception. Significant reduction in acidified saline‐induced nociceptive responses by 30–100 mg/kg, i.p. dose of myricetin (Córdova et al., 2011).

Not only for antinociceptive or neurological protection was tested myricetin, but also for its possible role as antimicrobial compound. As reported by Pasetto and collaborators myricetin could be effective against human papillomaviruses (HPVs) and human immunodeficiency virus (HIV). The polyphenol inhibited proliferation of infected cells with IC50 values of 3.23 lM (HIV‐1 89.6‐infected PBMC cells), 20.43 lM (HIV‐1 BaL‐infected TZM‐b1 cells), 4.49 lM (HIV‐1 MN‐infected PBMC cells), 22.91 lM (HIV‐1 MN‐infected H9 cells) and 1.76 lM (HIV‐1 89.6‐infected H9 cells) (Pasetto et al., 2014; Yuan et al., 2012). Similarly, studies on its antibacterial property revealed that it was effective against multidrug‐resistant *Burkholderia cepacia*, vancomycin‐resistant *Enterococci*, methicillin‐resistant *Staphylococcus aureus* and *Staphylococcus epidermidis* (Xu et al., 2016). DnaB helicase, a bacterial replicative factor, was found to be the main target for antibacterial activity of myricetin. DnaB helicase along with primase is the main part during DNA replication and elongation in *Escherichia coli* was noncompetitively inhibited (IC50 = 11.3 lM) by myricetin (Griep et al., 2007; Lin & Huang, 2012). Stimulation of the epithelial Cl‐secretion by myricetin was found usefulness in the prevention of viral/bacterial infection. Its effect was different from quercetin as this last could trigger epithelial Cl‐secretion merely under basal environment in epithelial A6 cells whereas myricetin could trigger it under basal as well as under cAMP‐stimulated conditions (Sun et al., 2014).

## 3 CONCLUSIONS AND FUTURE PERSPECTIVES

Myricetin shows great therapeutic potential especially against cancer, T2DM, liver injury, cardiovascular diseases, obesity and osteoporosis. These health benefits are proved by several in vitro and in vivo studies as previously reported. Convincingly, myricetin can induce apoptosis and can inhibit invasion, migration, adhesion along. It also interacts with several intracellular pathways, such as those related to insulin signaling, energy production. Moreover, infarct size and cardiomyocyte apoptosis levels were also found to be reduced by myricetin. Similarly, cytoprotective and inflammatory mediators have also been increased by myricetin, providing health benefits related to heart disorders and inflammation. Other studies suggested that hepatic biomarkers were modulated together with reduced oxidative stress and histopathological changes of the liver. Bone health was also demonstrated to be improved by myricetin, which was able to fight osteoporosis. In addition to these pharmacological activities, further studies especially based on a mechanistic approach are essential. These structure activity relationship (SAR) might be helpful to find a significant derivative of myricetin. Further, acute and chronic toxicological data on vital organs are of extreme importance. The clinical trial studies on myricetin are limited. According to a clinical survey, the consumption of myricetin can lead to low incidence of prostate cancer risk. Another clinical trial on lung cancer reported that the regular consumption of myricetin was associated with lung cancer decreased incidence. The consumption of myricetin along with other flavonoids by menopausal women resulted in a reduced risk of CHD (coronary heart diseases). The study also reported a dropping in sera TG, LDL and apolipoproteins. Moreover hyperglycemic levels were significantly normalized in T2DM patients. However, these clinical trials cannot be considered sufficient. Keeping in view the broad potential of this valuable compound, it is strongly recommended that other randomized double blinded clinical trials could be urgently conducted. Only after such research and deepen analysis, myricetin will be ready for market and we can look forward to a new tool for fight human diseases.

## CONFLICT OF INTEREST

There is no conflict of interest among authors.

## AUTHOR CONTRIBUTIONS

**Muhammad Imran:** Conceptualization (equal); Writing‐original draft (equal). **Farhan Saeed:** Writing‐original draft (equal); Writing‐review & editing (equal). **Ghulam Hussain:** Writing‐review & editing (equal). **ali imran:** Conceptualization (equal); Writing‐original draft (equal). **Zafar Mehmood:** Visualization (equal). **Tanweer Aslam Gondal:** Writing‐review & editing (equal). **Ahmad El‐Ghorab:** Conceptualization (equal); Writing‐original draft (equal). **Ishtiaque Ahmad:** Funding acquisition (equal). **Raffaele Pezzani:** Conceptualization (equal); Data curation (equal). **Muhammad Umair Arshad:** Conceptualization (equal); Writing‐original draft (equal). **Umar Bacha:** Conceptualization (equal); Writing‐original draft (equal). **Mohammad Ali Shariati:** Writing‐original draft (equal). **Abdul Rauf:** Conceptualization (equal); Writing‐review & editing (equal). **Naveed Muhammad:** Data curation (equal). **Gokhan Zengin:** Data curation (equal); Funding acquisition (equal). **Saiful Islam:** Conceptualization (equal); Writing‐review & editing (equal). **Zafar Ali Shah:** Writing‐review & editing (equal).

## Data Availability

Data available on request from the authors.
